# Preparation of honokiol-loaded titanium dioxide nanotube drug delivery system and its effect on CAL-27 cells

**DOI:** 10.3389/fbioe.2023.1249349

**Published:** 2023-08-03

**Authors:** Kaiqi Tang, Han Su, Zhi Qu

**Affiliations:** Second Affiliated Hospital of Jinzhou Medical University, Jinzhou, China

**Keywords:** titanium dioxide nanotubes (TNTs), Honokiol (HNK), tongue cancer, apoptosis, cell proliferation

## Abstract

**Background:** Tongue cancer is the most common type of oral cancer, and patients have a poor prognosis and quality of life after conventional surgical treatment. Honokiol (HNK) is a kind of lignan extracted from Chinese herbal medicine Houpu, many domestic and international experiments have demonstrated its anti-tumor effect. Titanium dioxide nanotube (TNTs) is a kind of nanomaterial which can be used as drug carrier. The purpose of this study is to explore the effects of HNK-loaded TNTs delivery system (HNK-TNTs) on anti-tumor.

**Methods:** TNTs were prepared by anodic oxidation method, and HNK was loaded onto TNTs by physical adsorption. The effect of HNK-TNTs on the proliferation, migration and apoptosis of CAL-27 cells were explored by CCK-8 experiment, scratch assay, live and dead staining and cellular immunofluorescence analysis.

**Results:** The material characterization test results showed that we had successfully prepared HNK-TNTs. CCK-8 experiment, scratch assay showed that the proliferation and migration ability of CAL-27 cells were significantly weakened after treatment with HNK-TNTs, and their cell proliferation rates significantly decreased. Live/dead staining, cell immunofluorescence analysis showed that HNK-TNTs could promote CAL-27 cells apoptosis by increasing the expression levels of the apoptosis-related protein Bax and Fas. Conclusion: In this experiment, we had successfully prepared Honokiol-loaded titanium dioxide nanotube drug delivery system (HNK-TNTs) and compared the effects of single drug HNK and HNK-TNTs on the proliferation, apoptosis and migration of tongue cancer CAL-27 cells. This experiment showed that HNK-TNTs had greater anti-proliferative, apoptosis-promoting and migration-inhibiting effects than the HNK as a single drug.

## 1 Introduction

Cancer is a common concern for people around the world, and oral cancer, which occurs in the mouth and jaw, is the eighth most common type of cancer (Zhou et al., 2020). Tongue, cheek and gum cancer are the three most common cancers in the oral cavity (Li et al., 2019). Tongue cancer is the most common type of cancer. The most common treatment for tongue cancer is mainly surgical, but after surgery, it greatly affects patients’ functions such as chewing, speech and swallowing, and the prognosis is poor, so the search for an effective anti-tongue cancer drug has become a hot research topic (Tang et al., 2020; Chang et al., 2022; Elseragy et al., 2022).

In recent years, using nanomaterials to deliver drugs has become a hot research topic, such as lipid-based materials (Lan, 2022). Titanium dioxide nanostructures (such as titanium dioxide nanotubes, titanium dioxide nanowires, etc.) can support a variety of drugs, such as anti-tumor drugs, bone proliferative drugs, anti-bacterial drugs, etc. (Akel et al., 2020; Mutalik et al., 2020). Titanium dioxide nanotubes (TNTs) have been widely used in anti-tumor clinical applications due to their good physical and chemical properties such as strong adsorption capacity. After drug loading, TNTs can play a nano role in tumor cells and improve the effect of a single drug on tumor treatment (Liao et al., 2020; Liu et al., 2022). In addition, Sun et al. proved that TNTs carrier has no effect on drug stability and is non-toxic and harmless to the body itself (Hasanzadeh Kafshgari et al., 2019; Sun et al., 2021).

**SCHEME 1:**
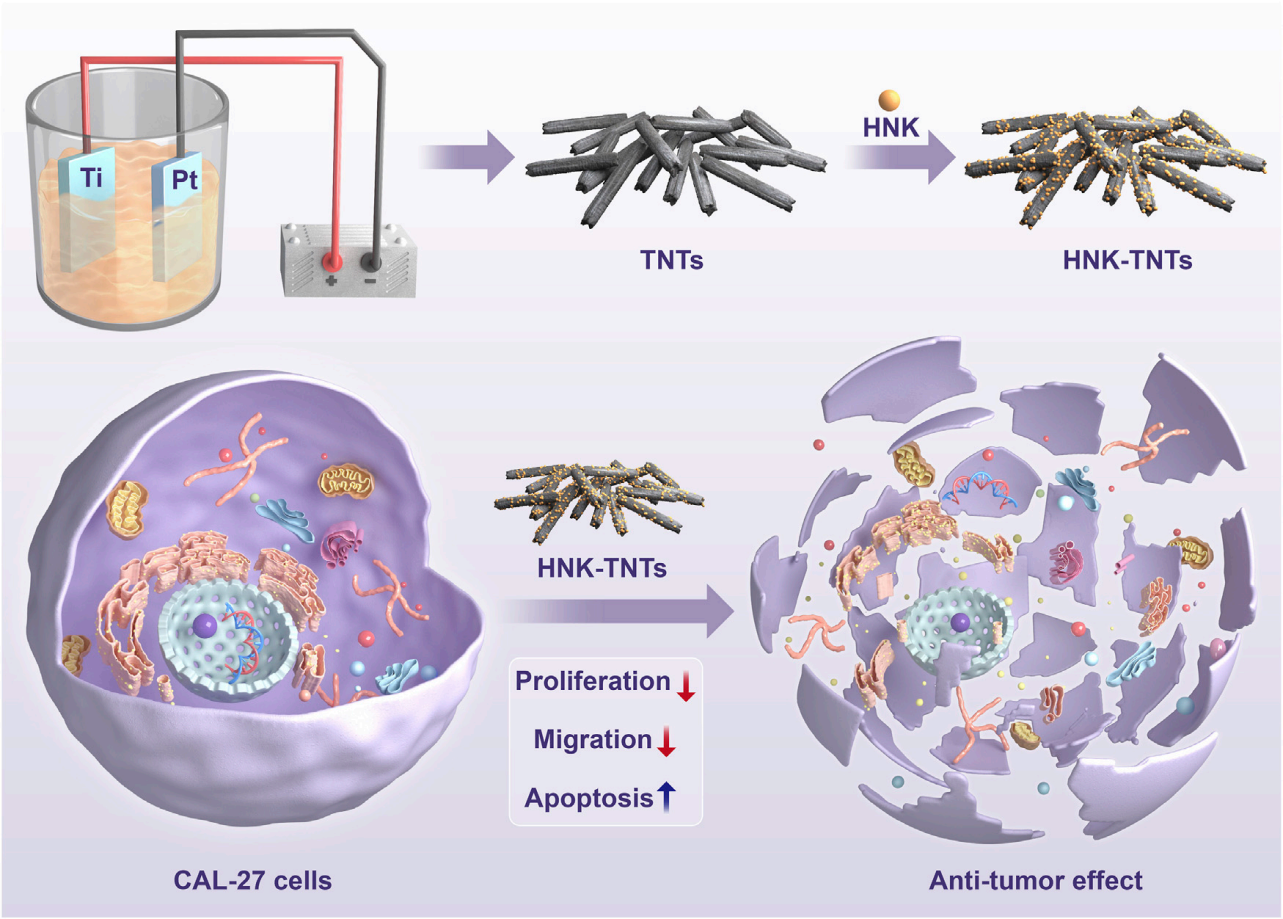
Preparation of HNK-TNTs and its anti-tumor effect on CAL-27 cells.

Honokiol (HNK) is one of the most important active ingredients in Chinese herbal medicine Houpu. Its biological activities and pharmacological effects include anti-tumor, anti-oxidant, anti-caries and anti-inflammatory (Liu et al., 2021; Lai et al., 2022). HNK has been shown to inhibit the growth of many cancer cells such as cervical cancer cells and lung cancer cells (Zhang et al., 2019; Zhang et al., 2022). In this experiment, TNTs were prepared by anodic oxidation and HNK was loaded onto TNTs by physical adsorption. It was shown that HNK-TNTs could enhance the ability of HNK to inhibit the ability of CAL-27 cells to multiply and migrate, HNK-TNTs promotes CAL-27 cells apoptosis at the same time. The efficacy on tongue cancer CAL-27 cells was explored to support future clinical applications (Paradowska et al., 2020; Asadi et al., 2022). The detail of the preparation process of HNK-TNTs and its mechanism of action on CAL-27 cells is shown in Scheme 1.

## 2 Materials and methods

### 2.1 Materials

Human tongue squamous cell carcinoma CAL-27 cell line was purchased from Shanghai Cell Bank, Chinese Academy of Sciences; Honokiol, purity: ˃98% was purchased from CSNpharm (United States); Fetal bovine serum was purchased from Clark Corporation (United States); DMEM culture-medium was purchased from Gibco (United States); 0.25% trypsin was purchased from Gibco (United States); DAPI was purchased from SouthernBiotech (United States); Cell Counting Kit-8 (CCK-8) kits were purchased from Sigma. Dimethyl sulfoxide (DMSO) was purchased from Solarbio (China).

### 2.2 Preparation of TNTs

The pure titanium sheets were ultrasonically cleaned with acetone, ethanol and de-ionized water and then dried at room temperature. Connected to a constant voltage power supply, oxidation was carried out in a glycol electrolyte (containing 0.3 wt% ammonium fluoride and 2 vol% water) at 50 V voltage and 1 h time, using the titanium plate as the anode and the platinum plate as the cathode. After oxidation, the surface was ultrasonically cleaned with deionized water and dried with high purity nitrogen to prepare TNTs.

### 2.3 Preparation of HNK-TNTs

A quantity of HNK powder was dissolved in anhydrous DMSO and the HNK solution was diluted to 40 μmol/L. The treated titanium flakes were placed in HNK solution, sonicated and shaken for 30 min. Rinse the titanium flakes with deionised water to remove excess impurities and store at room temperature in a dry place.

### 2.4 Materials characterization

The morphology and surface properties of the titanium dioxide nanotubes were observed using a field emission scanning electron microscope (SEM, ZEISS Gemini 300, Germany) and transmission electron microscope (TEM, JEM-1200EX, Japan). The crystalline structure of the TNTs and HNK-TNTs were analyzed by X-ray diffraction (XRD) (Bruker D2 Phaser). Fourier Infrared Spectroscopy (FTIR) (Thermo Scientific Nicolet iS20) was used to compare the composition of TNTs and HNK-TNTs.

### 2.5 Cell culture and disposal

Human tongue squamous cell carcinoma CAL-27 cells were cultured in DMEM medium containing 10% serum, 100 kU/L^−1^ penicillin and 100 μg/mL streptomycin and incubated at 37°C in a 5% CO_2_ incubator. Cells were allowed to grow to approximately 85% of the bottom of the dish for conventional passaging or subsequent cell experiments. The cells were divided into three groups:blank control group and HNK group and HNK-TNTs group.

### 2.6 Cell proliferation rate assay

Cell proliferation rate was assessed using the CCK-8 method. A density of approximately 5×10^4^ cells/well was inoculated into a 96-well plate. Appropriate concentrations of HNK and HNK-TNTs were respectively added into HNK group and HNK-TNTs group. After 1, 2 and 3 days incubation, 10 μL CCK-8 solution was added into each group and the incubation continued for 4 h 150 μL DMSO was added and the absorbance at 450 nm (A_450 nm_) was measured using an enzyme marker.

### 2.7 Cell scratch assay

A density of approximately 5 × 10^5^ cells/well of CAL-27 cells was inoculated into a 6-well plate and, after cell attachment, a linear scratch was created on the bottom of the well with a sterile pipette tip. The cell debris were washed for three times with PBS. Appropriate concentrations of HNK and HNK-TNTs were respectively added into HNK group and HNK-TNTs group. After 12 and 24 h incubation, cells were fixed with 4% paraformaldehyde, punched with 0.1% TritonX-100 (Sigma, United States) and stained with DAPI. Scratch healing after 12h and 24 h in different groups was observed by fluorescence microscope (Leica DM4000B, Germany), and the wound healing rate was calculated as follows: 12 h wound healing rate= (scratch area at 0 h-scratch area at 12 h)/scratch area at 0 h*100%; 24 h wound healing rate= (scratch area at 0 h-scratch area at 24 h)/scratch area at 0 h*100%.

### 2.8 Live/dead staining

CAL-27 cells at the logarithmic growth stage were inoculated into 24-well plates, and three compound pores were set in each group. After cell attachment, HNK and HNK-TNTs were respectively added into HNK group and HNK-TNTs group. After 24 h treatment, the supernatant was discarded, and the cells were stained by Calcein-AM/PI. Cells were cleaned with 1×Assay Buffer for 2–3 times, then 10 μL Calcein-AM and 15 μL PI solution were added into 5 mL of 1×Assay Buffer to prepare staining solution, and 200 μL staining solution was added to each well. The staining solution was removed after incubation at 37°C for 30 min in the dark. Different groups of live and dead cells were observed and photographed using fluorescence microscope (Leica DM4000B, Germany).

### 2.9 Immunofluorescence staining

CAL-27 cells at the logarithmic growth stage were inoculated into 24-well plates, and then the HNK group and HNK-TNTs group respectively treated with culture medium containing HNK and HNK-TNTs for 24 h. All groups’ cells were washed for three times with PBS, fixed with 4% paraformaldehyde for 30min and punched with 0.1% TritonX-100 (Sigma, United States). Anti-Tubulin (1:1,000), anti-Bax (1:200) and anti-Fas (1:200) primary antibodies were added and incubated at 4°C overnight, and the secondary antibody was added and incubated for 2 h after washing cells with PBS for three times. Then, the cells were stained with DAPI for 30 min. Finally, cell characterization was performed by fluorescence microscope (Leica DM4000B, Germany).

### 2.10 Statistical analysis

GraphPad Prism software, version 9 was used to create all charts in the paper. One-way analysis of variance (ANOVA) was applied to the analysis of statistical significances using *post hoc* Tukey’s multiplicative comparisons check. Differences among the groups were found to be statistically valid at *p < 0.05,**p < 0.01,***p < 0.001.

## 3 Results

### 3.1 Characterization of the materials

**FIGURE 1:**
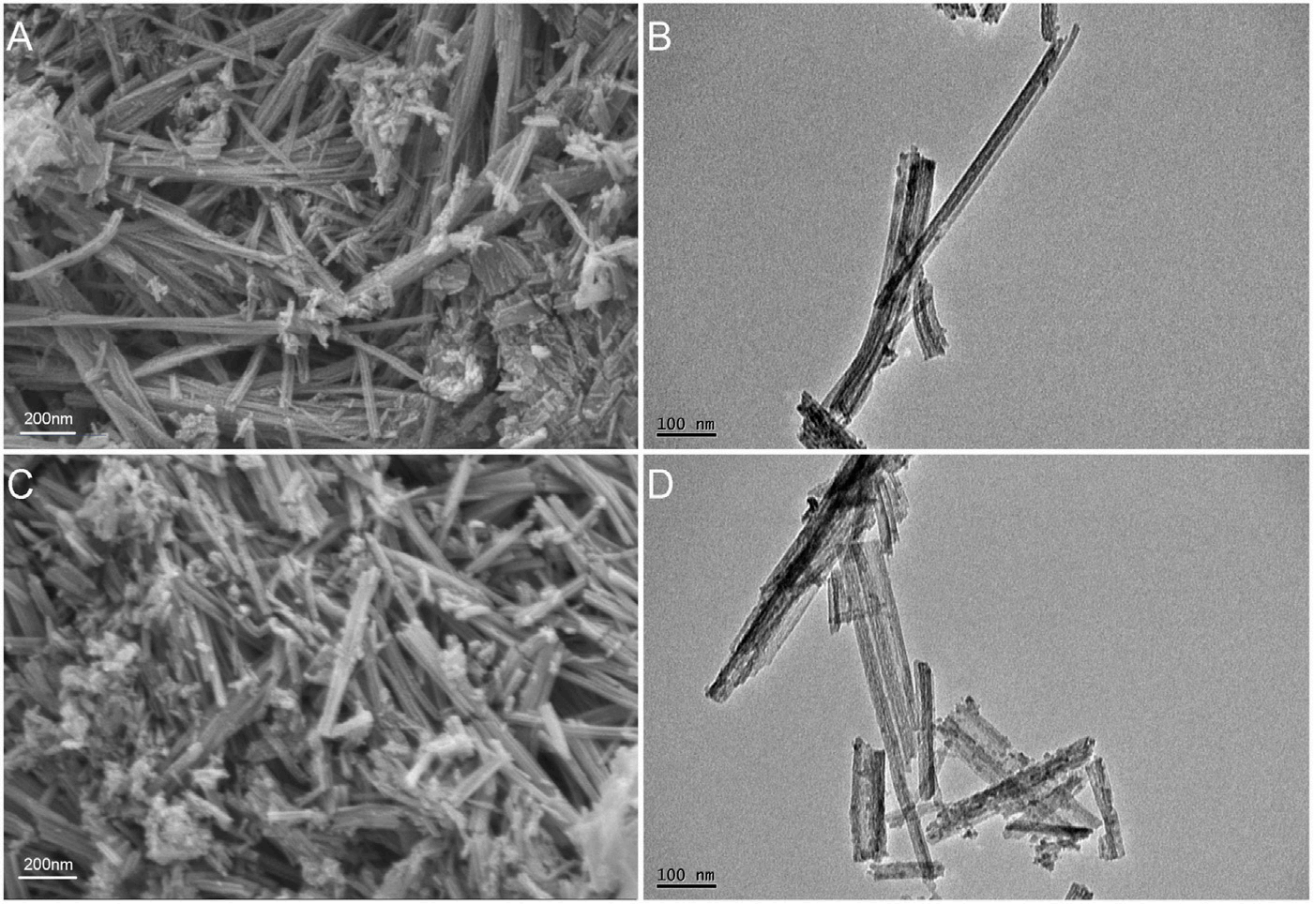
Material Characterization. **(A)** SEM image of TNTs. **(B)** TEM image of TNTs. **(C)** SEM image of HNK-TNTs. **(D)** TEM image of HNK-TNTs.

**FIGURE 2:**
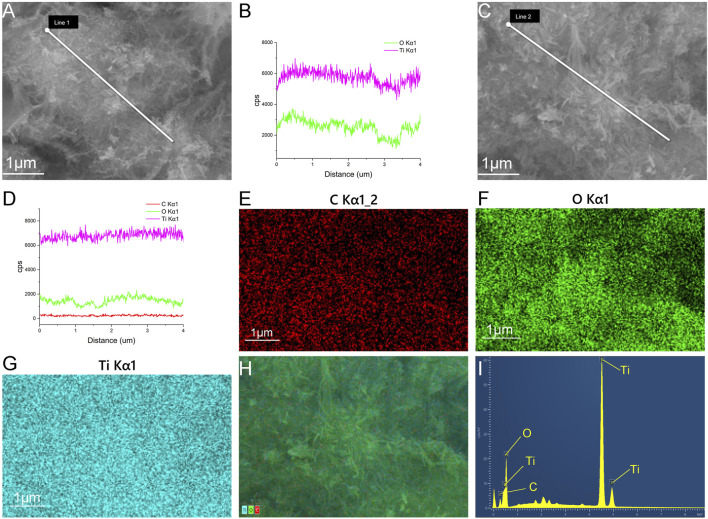
Material Characterization. **(A, B)** SEM line scanning results of TNTS. **(C, D)** SEM line scanning results of HNK-TNTs. **(E–H)** EDS mapping images of HNK-TNTs. **(I)** EDS spectrum of HNK-TNTs.

The SEM and TEM results showed that the TNTs had a uniform tubular structure with no sealing tubes at both ends (Figures 1A, B), so they were suitable for drug loading. After drug loading, the TNTs were coated around and inside with drugs, and the diameter of the tubes became rough (Figures 1C, D). It was preliminarily determined that HNK had been loaded on the TNTs. SEM line scanning results showed that, compared with TNTs, the introduction of element C indicated that HNK had been successfully loaded onto TNTs (Figures 2A–D); EDS mapping results showed that element C, O, and Ti were uniformly distributed in the prepared HNK-TNTs (Figures 2E–I), which is consistent with the results of SEM line scanning.

**FIGURE 3:**
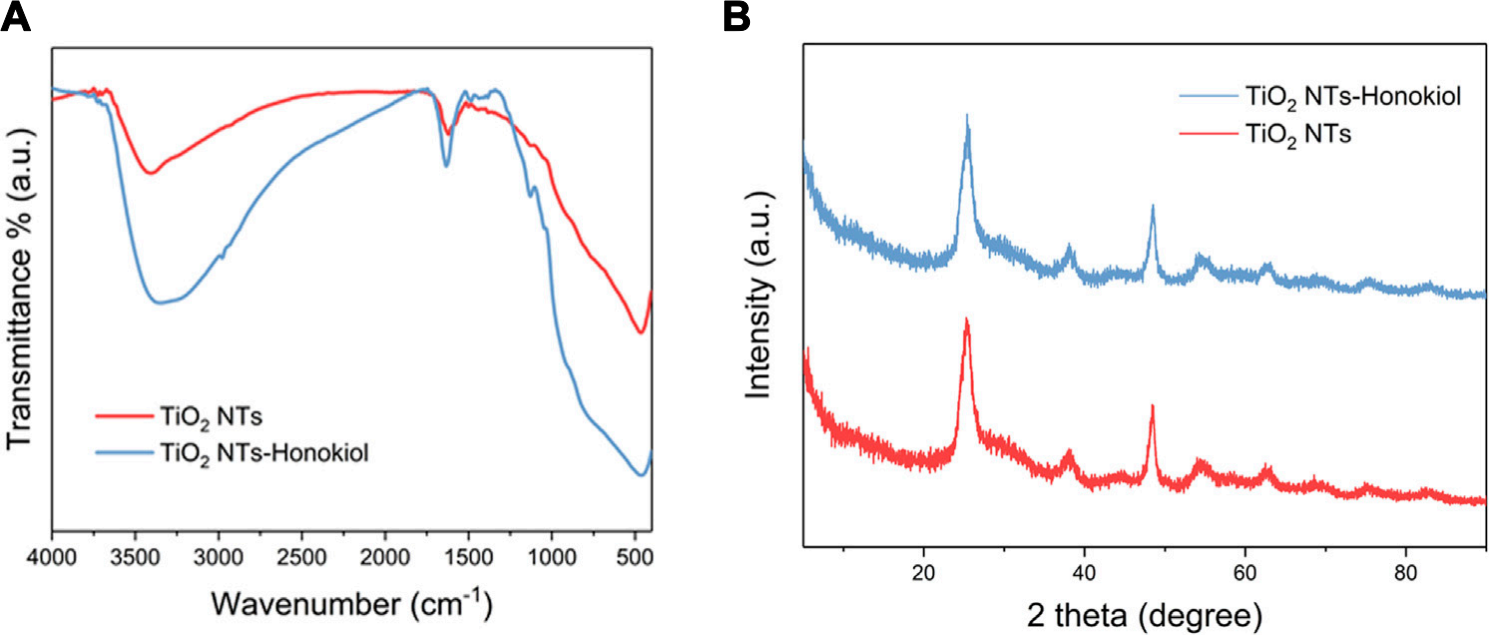
**(A)** FTIR spectra of TNTs and HNK-TNTs. **(B)** XRD patterns of TNTs and HNK-TNTs.

The resolution of FTIR test was 4.0 cm^−1^, and the scanning range was 4,000.0–400.0 cm^−1^. The absorption peak near 3,406 cm^−1^ indicated that hydroxyl groups existed on the surface of TNTs, and a large number of hydroxyl groups were the basis for HNK to be loaded onto TNTs. After HNK was successfully loaded, there was a significant difference between TNTs and HNK-TNTs in infrared spectrum, and the absorption peak density of hydroxyl groups decreased (Figure 3A), indicating that hydroxyl groups on the surface of TNTs did interact with HNK. This further supported our successful preparation of the HNK-TNTs. XRD results showed that there is no additional diffraction peak (Figure 3B), indicating that the loaded HNK has no effect on the crystal properties of TNTs.

### 3.2 Effect of HNK-TNTs on CAL-27 cell proliferation *in vitro*

**FIGURE 4:**
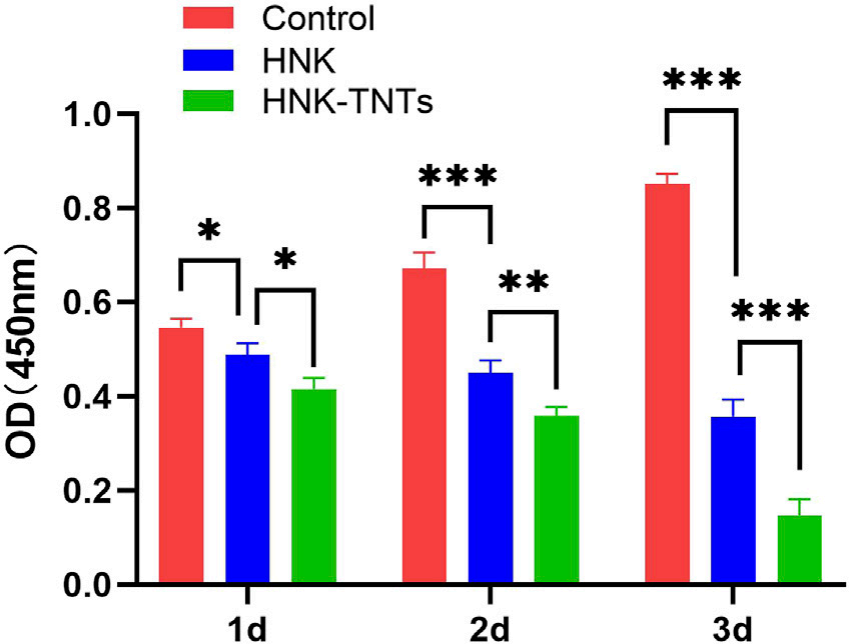
The CCK-8 results of different groups at different time. *p < 0.05,**p < 0.01,***p < 0.001*.*

The results of this CCK-8 experiment showed that compared with the control group, the proliferation rate of CAL-27 cells in group HNK and HNK-TNTs was negatively correlated with the time of drug action, and the proliferation rate in group HNK-TNTs was lower than that in group HNK (Figure 4), indicating that HNK-TNTs has a better effect on inhibiting CAL-27 cells proliferation than HNK.

### 3.3 Effect of HNK-TNTs on CAL-27 cell migration *in vitro*

**FIGURE 5:**
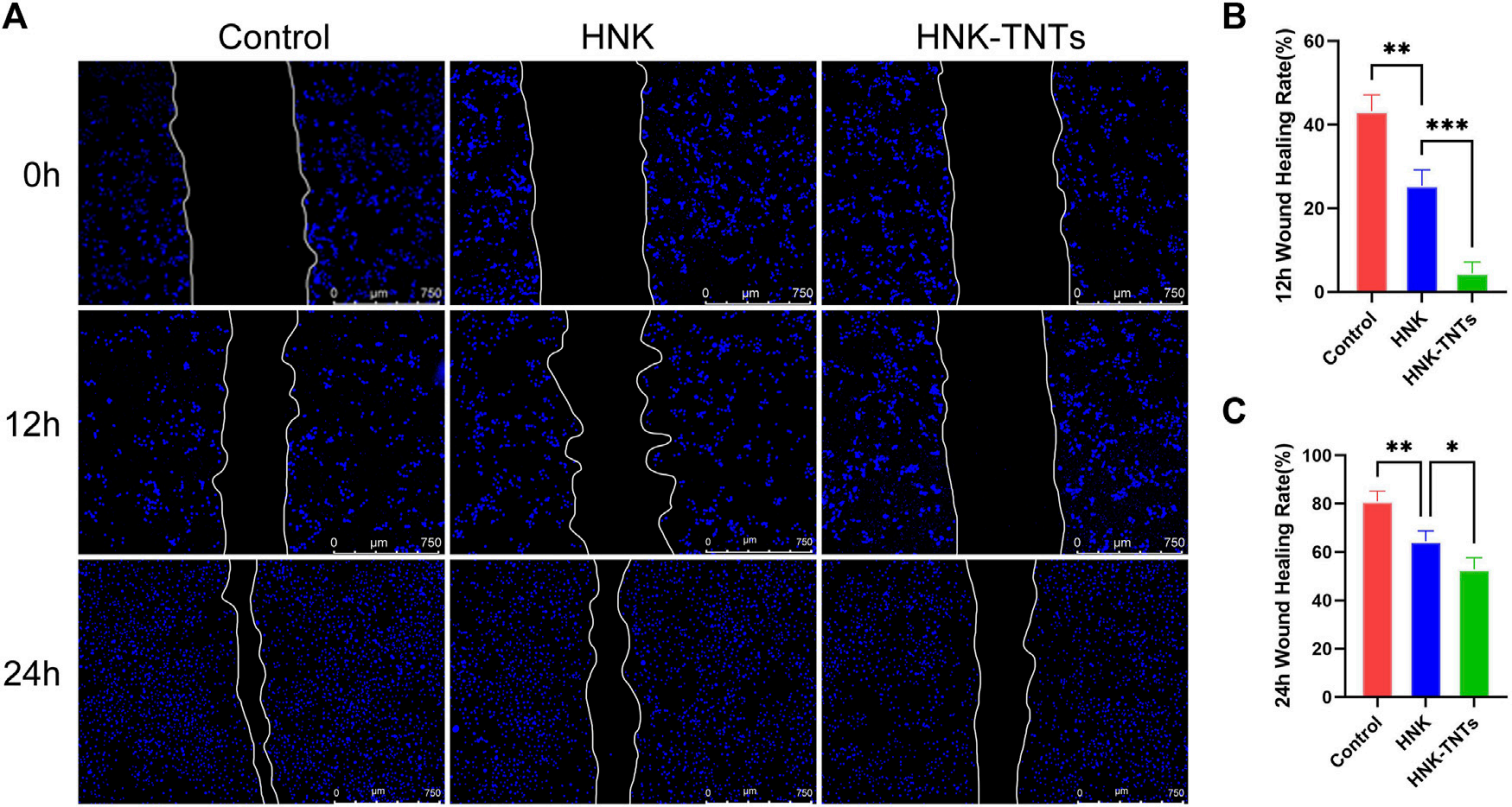
HNK-TNTs inhibited CAL-27 cells migration. **(A)** Photographs of the scratching experiment at different time. **(B, C)** Quantification of each time point for each group in the scratch experiment. *p < 0.05,**p < 0.01,***p < 0.001*.*

This scratching experiment results showed that after 12 and 24 h of cell culture, the migration ability of CAL-27 cells in group HNK-TNTs was significantly lower than in group control and group HNK (Figure 5A), and the difference was statistically significant (Figure 5B). It was shown that HNK-TNTs had a more significant inhibitory effect on cell migration.

### 3.4 Effect of HNK-TNTs on CAL-27 cell apoptosis *in vitro*

**FIGURE 6:**
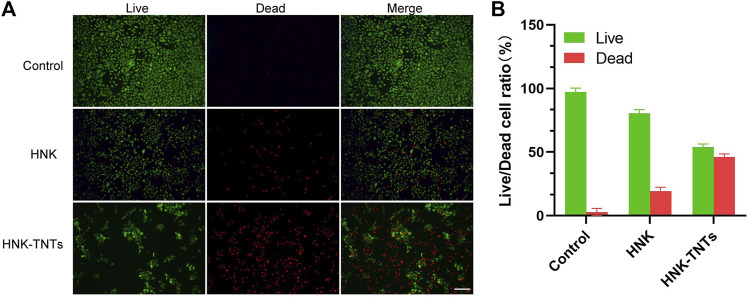
*In vitro* cell apoptosis assays. **(A)** Fluorescence images of live/dead staining of CAL-27 cells. Green: live cell staining; red: dead cell staining. bar = 100 μm.**(B)** Live/dead fluorescence staining for cell count analysis.

As shown in Figure 6, the control group only had very little dead cells. The ratio of dead cells in HNK group increased significantly, and the ratio of dead cells in HNK-TNTs group was higher than that in HNK group, indicating that HNK-TNTs had a more remarkable ability to promote CAL-27 cells apoptosis than HNK.

**FIGURE 7:**
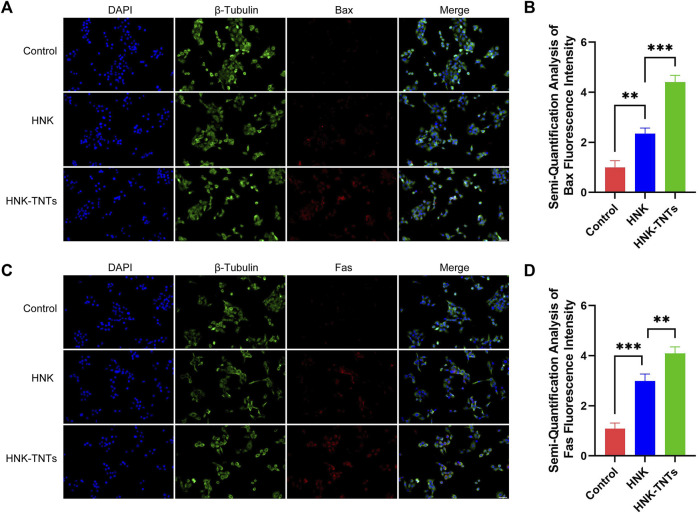
Mechanism of HNK-TNTs promoting the apoptosis of CAL-27 cells. **(A)** Immunofluorescence analysis of Bax protein expression. bar = 50 μm. **(B)** Fluorescence semi-quantitative analysis of Bax. *p < 0.05,**p < 0.01,***p < 0.001. **(C)** Immunofluorescence analysis of Fas protein expression. bar = 50 μm. **(D)** Fluorescence semi-quantitative analysis of Fas. *p < 0.05,**p < 0.01,***p < 0.001*.*

In order to further elucidate the mechanism of HNK-TNTs promoting the apoptosis of CAL-27 cells, we compared the expression of apoptosis factors Bax and Fas in different groups (Figures 7A, B). Semi-Quantification analysis of fluorescence intensity showed that the expression levels of Bax and Fas were significantly higher in the HNK-TNTs group than in the control and HNK group (Figures 7B, D). These results suggest that HNK-TNTs can promote apoptosis in CAL-27 cells by increasing the expression levels of Bax and Fas.

## 4 Discussion

Due to its stable chemical properties and good biocompatibility, titanium metal is widely used in the biomedical field, but it is found that titanium metal is bioinert in the application process, resulting in poor biological activity, so the modification of titanium has attracted a lot of scholars’ attention. In recent years, the use of nanomaterials to support drugs has become a research hotspot. At present, the common methods for preparing TNTs are: template synthesis, hydrothermal synthesis and electrochemical anodizing. Electrochemical anodizing is considered to be one of the most simple and effective methods for preparing TNTs (Indira et al., 2015). The process of preparing TNTs by anodic oxidation is to use titanium sheet as the anode, inert electrode such as platinum sheet as the cathode, and oxidize for a certain time in the electrolyte solution by means of DC voltage to form nanotubes. The specific principle is as follows: The formation process of TNTs is mainly a chemical reaction at the titanium/titanium oxide interface and the titanium oxide/electrolyte interface: Ti⇔Ti^2+^+2e^−^,2H_2_O⇔2O^2−^+4H^+^,2H_2_O⇔2O_2_+4H^+^+4e^−^,Ti^2+^+2O^2−^⇔ TiO_2_+2e^−^; In acidic electrolytes with fluoride ions present, the entire anodizing process is divided into the following four stagesTi^4+^+2H_2_O⇒TiO_2_+4H^+^: Titanium dissolves in the electrolyte and produces a large amount of Ti4+, and Ti4+ forms TiO2 films on the surface of Ti by interacting with oxygen-containing ions and water in the solution; TiO_2_+6F^−^+4H^+^⇒[TiF_6_]^2−^+2H_2_O: The TiO2 film is broken down and dissolved by the current to form depressed micropores; Micropores expand into small holes with the extension of time, and adjacent small holes fuse to form large holes, and then form a tubular structure; With the passage of time, the tubules gradually fuse into large tubules until the diameter becomes stable.

The TNTs can control drug release dynamics (Abdel Fadeel et al., 2021; Torres-Ramos et al., 2022). The various properties of TNTs, such as wettability and surface topography, can increase the surface area after drug loading. TNTs can reach the cell surface in two ways. One is the transcellular pathway, where the carriers are released into adjacent cells by transcellular translocation through pathways such as phagocytosis. The second is the paracellular pathway, which is the passive diffusion of nanocarriers (Barbara et al., 2021; Hernando et al., 2021; Peng et al., 2022).

Tumor cells are the process by which normal cells undergo genetic mutations in response to oncogenic factors. There are two types of tumor cells: benign tumor cells and malignant tumor cells. In general, benign tumor cells are not life-threatening, but malignant tumor cells can multiply indefinitely and become life-threatening. The CCK-8 results showed that HNK-TNTs could play an anti-tumor role by inhibiting the proliferation of CAL-27 cells.

Tumor metastasis refers to the movement of malignant tumor cells away from their original site (Leong et al., 2022). Tumor cells are transported through the blood and lymphatic system to adjacent organs and tissues where they can continue to multiply. As one of the important processes of tumor metastasis, cell migration is a complex regulatory mechanism. The scratching experiment results show that HNK-TNTs can impair the ability of tumors to metastasize by inhibiting cell migration.

Apoptosis is a complex regulatory mechanism divided into intrinsic pathway (the mitochondrial pathway) and extrinsic pathway (the death receptor pathway) (JRoberts et al., 2022). The mitochondrial pathway is also known as endogenous apoptosis pathway. Mitochondria are the main regulatory sites of apoptosis and participate in the regulatory process of most cell apoptosis (Vringer and Tait, 2023). Pro-apoptotic factors in mitochondrial membrane are released into the cytoplasm, activating the mitochondrial apoptosis pathway and causing cell apoptosis. Bax is one of the important apoptotic factors in this pathway (JoelRiley et al., 2018). The death receptor pathway is induced by the interaction of proteins mediated by extracellular signals through transmembrane receptors. The death receptor belongs to the tumor necrosis factor receptor (TNFR) superfamily, which contains cysteine-rich repeats outside the cell and a protein-binding domain of about 80 amino acid residues called the death domain (DD), which can bind specifically to its ligand to induce apoptosis. There are at least eight death receptors on the cell surface of the TNFR superfamily: Fas, TNFR1, TNFR2, DR3, DR4, DR5, DcR1 and DcR2, they are all members of the tumor necrosis factor α receptor family. The most typical of these death receptors are Fas and TNFRs (Fu et al., 2016). The results of immunofluorescence analysis showed that HNK-TNTs can induce CAL-27 cells apoptosis through Fas-mitochondria dual pathway.

Due to the limitation of experimental conditions, no animal model experiments were conducted in this study, and we will carry out animal model studies in due course to further verify the anti-tumor effect of HNK-TNTs.

## 5 Conclusion

In this experiment, we have successfully prepared HNK-TNTs. The material characterization test results show that HNK has been successfully loaded onto TNTs. The HNK-TNTs drug delivery system delivers HNK precisely to the inside of cells through surface molecular modification technology, enabling targeted drug delivery. This increases the aggregation of HNK in CAL-27 cells, thereby altering the metabolic kinetics of HNK. This experiment shows that HNK-TNTs significantly inhibits the proliferation and migration of CAL-27 cells. The HNK-TNTs drug delivery system promotes apoptosis in CAL-27 cells through upregulated levels of Bax and Fas. In contrast to the single drug HNK, HNK-TNTs drug carrier system is designed to improve efficacy, reduce side effects of drug molecules, maintain blood levels and promote inhibition of tumor cell migration and proliferation. In conclusion, the HNK-TNTs significantly inhibited the proliferation and migration, promoted the apoptosis of CAL-27 cells *in vitro*. This study provides an experimental basis for further clinical nanodrug delivery systems.

## Data Availability

The original contributions presented in the study are included in the article/supplementary material, further inquiries can be directed to the corresponding author.
